# Energy efficiency and carbon footprint assessment in artisanal and small-scale mining processing plants in Ecuador

**DOI:** 10.1007/s10653-025-02487-9

**Published:** 2025-05-02

**Authors:** Marcelo Moya, Javier Martínez-Gómez, Carla Carabajo, Angel Toapanta, Carlos Cruz

**Affiliations:** 1 Universidad de Alcalá (UAH), Escuela Politécnica, Departamento de Teoría de la Señal y la Comunicación, (Área de Ingeniería Mecánica), Alcalá de Henares, 28801 Madrid Spain; 2 Facultad de Ciencias Técnicas, Universidad Internacional del Ecuador UIDE, 170411 Quito, Ecuador; 3 Instituto de Investigación Geológico y Energético (IIGE), 170518 Quito, Ecuador; 4 Facultad de Arquitectura e Ingenierías, Universidad Internacional SEK UISEK, Albert Einstein S/N and 5th, 170302 Quito, Ecuador

**Keywords:** Energy consumption, Gross energy requirement (GER), Energy intensity index (EII), Sustainable mining practices, CO_2_ emissions

## Abstract

Artisanal and small-scale mining (ASM) plays a significant role in global mineral production, particularly in developing countries, providing employment and supporting local economies. However, ASM is characterized by high energy intensity, inefficient processing technologies, and a substantial carbon footprint, primarily driven by reliance on electricity and diesel-powered machinery. This study evaluates key energy indicators including Gross Energy Requirement, Energy Intensity Index, and CO_2_ emissions to identify patterns and inefficiencies in energy consumption across ASM operations. Findings reveal that grinding processes account for the highest energy consumption, while electricity contributes over 99% of total energy usage, generating an average emission factor of 25.55 kg CO_2_/GJ from 2019 to 2023. Diesel and GLP, though minor in contribution, remain critical sources of emissions. The analysis underscores the strong correlation between energy intensity and CO_2_ emissions, highlighting the environmental impact of operational inefficiencies. Recommendations include adopting energy-efficient technologies, optimizing energy management systems, and enhancing regulatory frameworks to reduce energy consumption and minimize the sector's carbon footprint. This study provides actionable insights for policymakers and stakeholders to balance economic benefits with environmental sustainability in ASM practices.

## Introduction

Artisanal and small-scale mining (ASM) is a significant global activity, contributing up to 20% of the world’s gold production and providing livelihoods for roughly 100 million people, including 15 million miners—many of whom are women and children (Keane et al., 2023). However, ASM often operates with outdated technologies and limited technical expertise, which drive high energy consumption and operational inefficiencies (Mestanza-Ramón et al., 2022). These inefficiencies heighten environmental impacts especially relevant to sustainability goals and restrict socio-economic benefits in areas where ASM could otherwise support local development (Alves et al., 2021). Although ASM can yield substantial economic gains for rural communities, it also presents considerable energy, environmental, and social challenges. Gold mining processes are often marked by inefficient grinding and other suboptimal practices (Gaudry et al., 2020; Mulaba-Bafubiandi et al., 2023). Consequently, targeted interventions that foster the adoption of energy-efficient techniques and technologies are vital for reducing environmental degradation and enhancing the livelihoods of those dependent on ASM.

Historically, academic efforts have centered on two thematic areas: (1) the environmental and social consequences of mining activities, and (2) mining’s positive socio-economic contributions (Alves et al., 2021). Mineral extraction is a vital driver of employment, supply-chain value, and industrial competitiveness, aligning it closely with several United Nations Sustainable Development Goals (SDGs) (Fuso Nerini et al., 2024; Nations, 2023). At the same time, mineral production can undermine other SDGs by contributing to environmental degradation, compromised water resources, and health risks (Mancini & Sala, 2018).

Methodological approaches across these studies range from quantitative assessments, such as life-cycle analysis (Helling, 2017; Islam et al., 2020) and energy audits (Abbaspour & Abbaspour, 2022; Aramendia et al., 2023; Purhamadani et al., 2021), to qualitative case studies examining social and policy dimensions (Clifford, 2022; Li et al., 2023). For instance, researchers often track specific energy indices (e.g., energy intensity, comminution energy consumption) alongside interviews and field observations (Martins et al., 2022), enabling a nuanced understanding of how technological limits and policy gaps manifest in real-world ASM conditions (Famiyeh et al., 2021). Although these metrics and frameworks differ in scope—ranging from micro-level data collection at individual processing plants to macro-level policy evaluation—they collectively underscore the multidimensional nature of sustainability challenges in ASM. Such variability in methods, however, also amplifies the need for standardization to facilitate cross-comparisons and inform evidence-based policymaking.

Within ASM, a substantial portion of studies has examined energy-intensive processes, especially comminution (crushing and grinding), which are key contributors to greenhouse gas emissions (Gaudry et al., 2020; Jeswiet & Szekeres, 2016). Research approaches commonly include: (1) case studies quantifying on-site energy consumption and highlighting areas for potential optimization (Avalos et al., 2020; Jeswiet & Szekeres, 2016; Katta et al., 2020; Marsden & House, 2006; Pereira et al. 2021; Purhamadani et al., 2021); (2) techno-economic analyses of integrating renewable energy sources (Ansong et al., 2017; Igogo et al., 2021; Jacob & Müller, 2022; Ostrowski et al., 2015); (3) policy reviews that stress the importance of stronger regulations and improved environmental oversight (Efendieva & Khalifazade, 2018); and (4) systems modeling to estimate the broader socio-economic implications of increased energy efficiency (Aramendia et al., 2023).

Recent case studies in regions like southwestern Ghana leverage field-based observational and empirical research to assess ASM’s contribution to elevated CO_2_ emissions and environmental degradation. These investigations often combine on-site energy consumption measurements with surveys of local practices, revealing the ramifications of diesel dependency and mercury-based gold extraction on greenhouse gas emissions and biodiversity loss (Barenblitt et al., 2021; IPCC, 2006). The unregulated nature of artisanal mining characterized by minimal governmental oversight further exacerbates environmental harm, prompting calls for mixed-method assessments that integrate policy analysis, stakeholder interviews, and geospatial data (Morante-Carballo et al., 2022). Findings generally converge on the need for reinforced regulatory frameworks, targeted energy-efficient technologies, and community-focused educational programs to balance socio-economic benefits with ecological sustainability (Clifford, 2022; Saka et al., 2024).

A parallel stream of research employs econometric modeling and policy analysis to evaluate how carbon pricing, emissions trading mechanisms, and digital trade initiatives shape the mining sector’s environmental footprint (Wang et al., 2023). Studies by Green (2021); (Grubb et al., 2021) and others apply comparative, cross-country evaluations, highlighting that higher carbon market prices spur cleaner production methods and catalyze low-carbon technological innovation (Lilliestam et al., 2021; W. Zhang et al., 2022). Methodologically, these inquiries rely on macro-level data sets often drawn from international markets and national economic reports to explore how businesses respond to varying regulatory pressures, carbon tax rates, and economic incentives (Finn et al., 2024; S. Li & Shao, 2023; Missbach et al., 2024). They collectively underscore the importance of robust legal frameworks, transparent monitoring systems, and consistent global alignment to ensure that both developed and developing mining regions can effectively integrate carbon reduction strategies.

Artisanal and small-scale mining (ASM) is a significant economic activity in Ecuador, playing a crucial role in the country’s mining sector while also posing substantial environmental challenges. ASM operations are prevalent across various regions in Ecuador, each with its distinct mineral deposits. The coastal areas are rich in non-metallic minerals and gold placers, while the highlands are known for their limestone and metallic/non-metallic mineral resources. The Amazon region, in particular, is abundant in valuable minerals such as gold, silver, copper, antimony, and has the potential for lead, zinc, and silica sand extraction (Prodeminca, 2000). Among these, gold stands out as the most extracted underground mineral, with 8.21 tons produced in 2018 alone, generating significant revenue of $526 million (Secretaria Nacional de Planificación, 2021). Additionally, the production of non-metallic minerals reached 5.84 million tons, contributing $4.31 million, and highlighting the sector’s contribution to Ecuador’s GDP at 1.63% in 2018.

In 2020, the provinces of Zamora Chinchipe, Morona Santiago, El Oro, Azuay, and Loja were identified as the most active in mining, collectively hosting 924 of the 1458 mining concessions in Ecuador. Notably, 76.40% of these concessions were operated by small-scale and artisanal miners (MERNNR, 2020). Despite its economic significance, the ASM sector faces numerous challenges, including outdated technology, insufficient training, inefficient energy use, and difficulties in adhering to regulatory frameworks (Oviedo-Anchundia et al., 2017). Furthermore, studies have indicated that water quality in some mining areas does not meet regulatory standards, and the introduction of new processing plants in industrial zones has led to issues such as power line saturation and voltage instability (IIGE, 2020, 2021).

The environmental impact of ASM operations is particularly pronounced in the Zaruma-Portovelo area, where processing plants use mills and cyanide leaching techniques for mineral extraction. These processes produce toxic tailings containing heavy metals, posing a significant risk to the environment and public health (Oviedo-Anchundia et al., 2017). Mercury contamination, a byproduct of gold extraction, remains a critical concern, with attempts to mitigate the environmental damage often leading to unintended consequences (Gaudry et al., 2020). The sharp increase in global gold prices between 2002 and 2012 fueled a surge in production, which has continued, with mining exports increasing by 106.96% in early 2023 (Banco Central del Ecuador, 2023). However the National Energy Balance 2022, the reported that mining, grouped with agriculture and fishing, represented only 1.2% of Ecuador's total energy consumption, a figure that notably excludes the actual energy use by ASM operations (Instituto de Investigación Geológico y Energético, 2022a).

Ecuador’s energy crisis, characterized by frequent electricity shortages and instability, further complicates the challenges faced by the ASM sector. The country's reliance on hydropower, which is vulnerable to climate variability, has led to periodic disruptions in electricity supply. This instability is particularly problematic for energy-intensive industries like mining, where consistent and reliable power is determinant for operations. The ASM sector, often operating in remote areas with limited infrastructure, is especially vulnerable to these disruptions. The reliance on fossil fuels for energy, in the absence of reliable electricity, exacerbates environmental impacts and increases operational costs.

This research makes a substantial contribution to understanding energy consumption in artisanal and small-scale mining (ASM) processing plants, particularly in gold and copper extraction. By introducing a novel methodology for calculating energy indicators and analyzing production cycles, this study builds upon and refines existing approaches (Aramendia et al., 2023; Gaudry et al., 2020). The analysis incorporates data from seven mining companies between 2019 and 2023, focusing on technical parameters such as operating permits, equipment registration, and energy consumption records. Key energy indicators, including Gross Energy Requirement (GER), Energy Intensity Index (EII), and CO_2_ emissions, are assessed to identify inefficiencies and trends in energy use. The study highlights grinding processes as the most energy-intensive activity, with electricity accounting for over 99% of total energy consumption and generating an average emission factor of 25.55 kg CO_2_/GJ. Diesel and GLP, though minor contributors, remain significant sources of emissions. Additionally, the impact of international market price fluctuations on mineral production is explored. Through quantitative analysis and statistical modeling, the findings underscore the strong correlation between energy intensity and CO_2_ emissions, emphasizing the environmental consequences of operational inefficiencies. This research offers actionable recommendations, including the adoption of energy-efficient technologies, optimized energy management systems, and strengthened regulatory frameworks. These insights provide a foundation for sustainable resource management and policy development, aiming to improve energy efficiency and operational sustainability in the ASM sector while balancing economic benefits with environmental responsibility.

Given the context of Ecuador’s energy crisis, this research holds relevance by addressing the urgent need for improved energy efficiency in artisanal and small-scale mining (ASM) operations. Enhancing energy practices in this sector can significantly reduce reliance on an unreliable electricity supply, thereby mitigating the cascading effects of energy instability on the national economy. By identifying inefficiencies, such as the high energy intensity of grinding processes and the disproportionate contribution of electricity to overall energy consumption, this study highlights actionable opportunities to optimize resource use and decrease the sector's carbon footprint. Moreover, implementing the recommended energy-efficient technologies and management systems could support national efforts to stabilize the energy grid, reduce CO₂ emissions, and promote more sustainable economic development. These insights are pivotal not only for the ASM sector but also for advancing Ecuador’s broader energy policies and achieving sustainable development goals, positioning the country as a model for balancing economic growth with environmental responsibility.

## Methodology

**Fig. 1:**
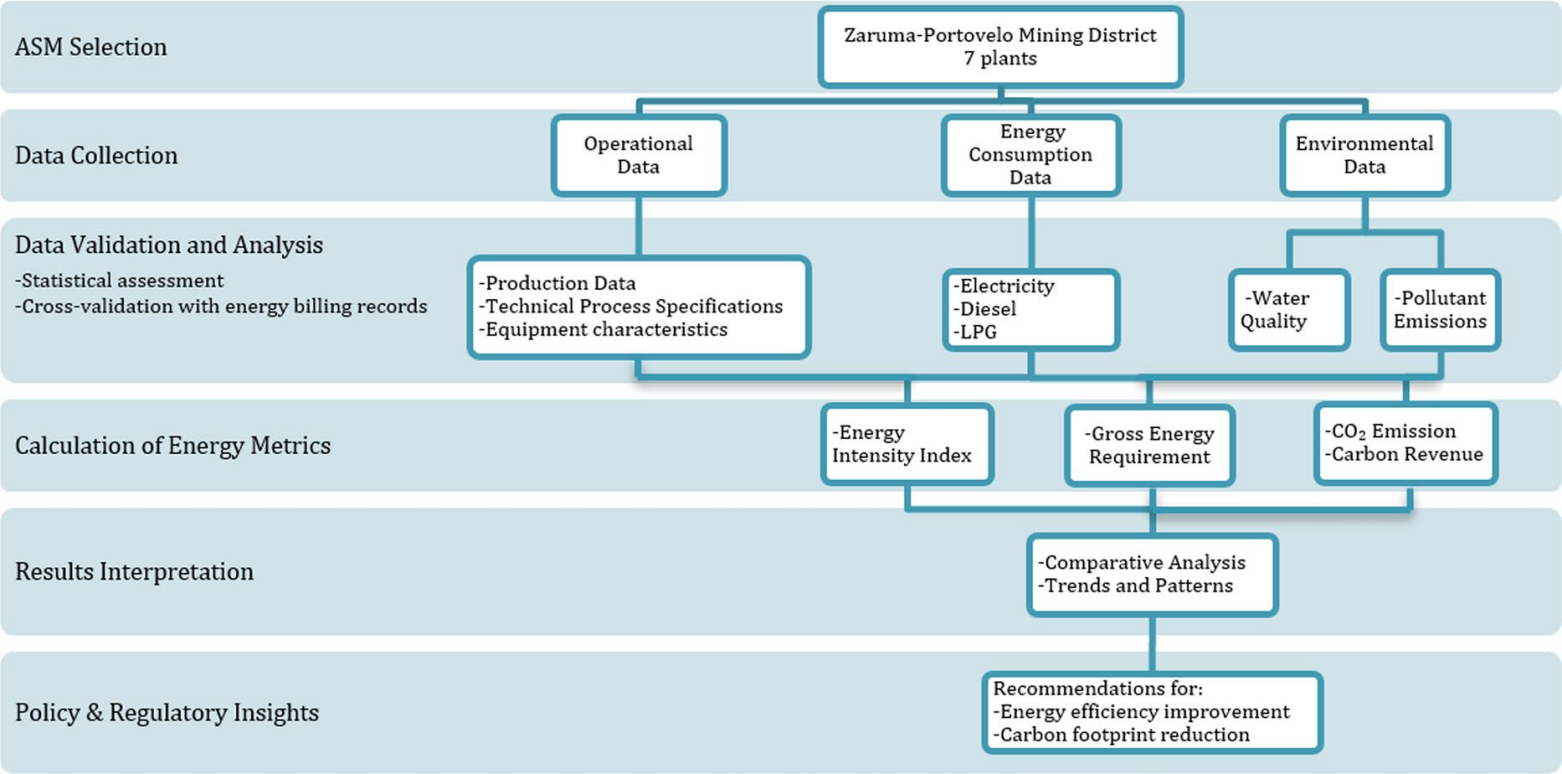
Methodological process description

The study aims to provide a comprehensive understanding of energy use within these operations, particularly in the context of gold and copper extraction. The methodological framework, which includes data collection, technical parameter evaluation, and quantitative analysis for assessing energy consumption in artisanal and small-scale mining (ASM) processing plants within Ecuador's Zaruma-Portovelo mining district, is depicted in Fig. 1. In the theoretical analysis of the baseline of energy consumption in the Zaruma-Portovelo mining district all mining operations(underground mines) and gold processing facilities (utility plants, relief deposits) were identified in order to determine their energy consumption (Instituto de Investigación Geológico y Energético, 2022b). Gaudry et al. (2020) propose the general initial sample of 74 potential mineral processing plants in Zaruma-Portovelo, their study served as a starting point to develop research and technical visits in 7 companies where they have historical records for the period 2019–2023.

### Energy and mining data collection

The data collection for this study was executed through two key projects: “Improving Working Conditions in Small-Scale and Artisanal Mining” and “Study to Determine Active and Passive Energy Efficiency Strategies in Mines and Gold Processing Plants in the Zaruma-Portovelo Mining District”. These initiatives were fundamental in providing a comprehensive historical evaluation of electricity and fossil fuel usage in selected mining companies from 2019 to 2023. By employing detailed methodologies, the projects effectively monitored transformation capacity and established a baseline dataset. This dataset covered critical aspects such as production, energy consumption, water quality, and pollutant emission controls, which were meticulously disaggregated by each process and subprocess involved in the extraction and processing of gold and copper.

The data collection process was conducted through a collaborative approach involving technicians from the Institute and personnel from the participating mining companies. This collaboration ensured that the collected data accurately represented the operational conditions prevalent within artisanal and small-scale mining (ASM) processing facilities. Employing this comprehensive and participatory methodology established a robust empirical basis for subsequent analysis, thereby enhancing the practicality and applicability of the study's findings to real-world scenarios within the ASM sector.

One of the key aspects of data collection was the detailed information gathered on mineral extraction from underground mines, particularly focusing on the amount of material processed in benefit plants. The data was carefully disaggregated by specific processes and subprocesses, providing an in-depth view of the steps involved in gold and copper extraction. For energy consumption, the study used Class A energy quality analyzers, as defined by the IEC 61000-4-30 standard, to perform precise field measurements in the ASM processing plants. The accuracy of this data was further corroborated with energy consumption records provided by the local electricity distribution company, CNEL, ensuring a comprehensive and reliable understanding of energy usage patterns.

The dual-method approach, integrating direct field measurements with utility-provided data, ensured a high level of confidence in the accuracy and reliability of the collected energy consumption information. Utilizing Class A energy analyzers, renowned for their rigorous precision standards, was necessary in verifying and validating the recorded energy usage. This meticulous approach to data collection significantly enhanced the credibility of the subsequent analytical outcomes and provided a robust foundation for formulating targeted policy recommendations to improve energy efficiency within the mining sector.

Furthermore, the projects emphasized monitoring water quality and controlling pollutant emissions, with data disaggregated by each process and subprocess within the extraction and processing operations. This environmental monitoring was central for assessing the impact of ASM activities and developing strategies to mitigate negative effects. The comprehensive data collection strategy established by these projects thus provided a robust basis for evaluating energy efficiency and environmental sustainability in the ASM sector, ultimately helping identify opportunities to optimize energy use and improve the sustainability of mining operations in the Zaruma-Portovelo district.

### Underground mines and benefit plants

*Underground Mines* These facilities involve the extraction of gold deposits from beneath the Earth's surface through drilling, blasting, and excavation. They require extensive infrastructure, including transportation systems, ventilation, and safety measures.

*Benefit Plant* These facilities process extracted gold to separate valuable minerals from waste materials using techniques such as crushing, grinding, washing, and chemical treatment. The goal is to enhance the quality and purity of the minerals, making them suitable for further processing or market sales.

Underground mines involve the extraction of gold deposits from beneath the Earth's surface, typically through drilling, blasting, and excavation. These mines access mineral deposits buried deep underground, requiring extensive infrastructure for transportation, ventilation, and safety measures. Benefit plants are facilities where extracted gold undergoes processing to separate valuable minerals from waste materials. This process involves various techniques such as crushing, grinding, washing, and chemical treatment to concentrate the desired minerals and remove impurities. Benefit plants aim to enhance the quality and purity of extracted minerals, making them suitable for further processing or market sale. The nomenclature and characteristics of the 7 selected companies are presented in Table 1., (Instituto de Investigación Geológico y Energético, 2022b).

**Table 1:** Companies characteristics

Operation	Coordinate UTM	Characteristics
Plant 1	652111, 9590419	Processing plant that only rents its services to process material from the area The plant processes treatment of the gold is Cyanidation gold bars
Plant 2	653103, 9594561	Processes the material obtained from Mine 2 and provides rental services to external clients The plant carries out two processes for the treatment of the gold. (a) Cyanidation gold bars (b) Flotation copper and silver concentrates
Plant 3	652114, 9589781	Processing plant that only rents its services to process material from the area The plant processes treatment of the gold is Cyanidation gold bars
Plant 4	652049, 9589152	Processing plant that only rents its services to process material from the area The plant processes treatment of the gold is Cyanidation gold bars
Plant 5	650279.96, 9593777.58	Processes the material obtained from Mine 1 The plant carries out two processes for the treatment of the gold. (a) Cyanidation gold bars (b) Flotation copper and silver concentrates
Mine 1	654000, 9592740	The material extracted is processed in the benefit plant 5
Mine 2	653100, 9593777.58	The material extracted is processed in the benefit plant 2

The selection of technical parameters in this study was essential for gaining a comprehensive understanding of the energy dynamics and operational characteristics of artisanal and small-scale mining (ASM) processing plants in Ecuador. These parameters were chosen based on their relevance to key aspects such as energy consumption patterns, operational efficiency, regulatory compliance, and environmental impact. Operating permits and company classification provided insights into the legal and regulatory framework within which ASM operations are conducted, ensuring that the study considered the institutional context of these activities. Equipment registration and P&ID (Piping and Instrumentation Diagrams) diagrams, on the other hand, offered a detailed understanding of the processing plant infrastructure, enabling a thorough analysis of how equipment and processes contribute to energy consumption.

Location data and compliance with electrical standards are important for contextualizing the operational environment and ensuring safety and reliability. Location information helped assess environmental and logistical considerations, while adherence to electrical standards confirmed the integrity and safety of the energy infrastructure in these facilities. Historical production data and energy consumption records were particularly valuable, allowing for an analysis of past performance and the identification of areas where efficiency improvements could be implemented. These records, combined with water consumption data, helped build a comprehensive picture of resource utilization in ASM processing plants.

By integrating these parameters, the study could construct a thorough baseline of production and energy use in the ASM sector, enabling informed decision-making and policy development. The careful selection of parameters ensured that all aspects of the mining process were evaluated, from legal compliance to operational efficiency and environmental sustainability. This comprehensive approach not only enhances the understanding of energy utilization in ASM processing plants but also provides valuable insights for developing strategies to improve energy efficiency and promote sustainable resource management in the mining sector.

### Quantitative analysis and energy consumption indicators

A multidimensional approach was employed to evaluate key energy consumption indicators, including the Gross Energy Requirement (GER), Energy Intensity Index (EII), and treated tonnage. The study also analyzed the impact of international market price fluctuations on gold and copper production cycles, using statistical models to identify patterns and trends in energy use. These insights are crucial for optimizing energy utilization and enhancing operational efficiency in ASM processing plants.

### Historical final energy consumption of the mining industry

After obtaining the aforementioned data, we used the methodology presented in the work of Aramendia et al. (2023) to determine the energy consumption profiles for each gold-processing plant and mining operations, the historical final energy consumption (measured in joules) for a specific mineral may be found using the following Eq. 1:

1 $$EII=\frac{EC}{TM}$$

where EII (Joule/tons) is the Energy Intensity Index, EC (Joule) is total Energy Consumed (diesel oil, electricity and liquefied petroleum gas GLP) and TM (ton) is the treated tonnage of gold.

The quantity of energy required to provide a specific level of output or activity is known as energy intensity. Reduced energy intensity is the outcome of using less energy to provide a good or render a service (“Energy Intensity Indicators Methodology, Caveats, and Cautions”, 2024). Methodology of calculation for energy intensity is computed by dividing the annual energy consumption of the machinery (in kBtu or GJ) by the total production per year (in tons) (Energy, 2024).

### Historical energy intensities by extracted mineral

Mining activities require direct and indirect energy, which are quantified using Life Cycle Analysis methods (The Gross Energy Requirement (GER) indicator is called the Cumulative Energy Demand (CED) in the Life Cycle Analysis terminology). Direct energy requirements are used in situ to operate the mine, while indirect energy requirements are used ex-situ in the mine supply chain to provide inputs. The Gross Energy Requirement (GER) is obtained by adding both direct and indirect energy requirements. As mining energy requirements are likely to increase due to mineral depletion, GER is used to measure the primary energy intensity of each mineral (Nuss & Eckelman, 2014; Tabelin et al., 2021).

$${GER}_{year}=\sum _{monthly}Electric ~Energy+Diesel ~oil ~Energy+Petroleum~ liquid ~gas$$

### Energy consumption per process

The methodology for assessing energy consumption in mining operations involved the deployment of high-precision energy analyzers to capture detailed data on electricity usage. Devices such as the Sonel PQM 711, Fluke 1760, Fluke 1777, and Fluke 435 series II were employed in accordance with the IEC 61000-4-30 standard, ensuring accurate and comprehensive measurements. These analyzers were strategically installed at key points within the mining facilities, including main panels, low-voltage transformer outputs, and panels dedicated to compressors, crushers, grinding mills, and flotation cells in benefit plants. This allowed the study to target areas of highest consumption, which are critical for understanding overall energy use.

Data collection was conducted over an extended period of 30 days, with measurements recorded at 10 min intervals to capture fluctuations and variations in consumption patterns. This high-frequency sampling ensured that the energy consumption data reflected both the steady-state and peak demands of different mining processes, providing a robust basis for analyzing efficiency across operations. Plant 2 and Plant 3 were chosen for the study because they exhibited the highest energy consumption within the mining district, making them representative of the most energy-intensive processes in small-scale mining operations. Focusing on these plants allowed the study to capture detailed data on critical processes like crushing, grinding, and flotation, providing insights into areas where energy efficiency improvements would be most impactful. Their inclusion ensured that the findings could be applied to similar mining facilities, enhancing the relevance and applicability of the results.

Analyzing both plants allowed for a comparative assessment of energy consumption across different operational setups. This comparison provided valuable insights into best practices and inefficiencies unique to each plant, establishing benchmarks for optimizing energy usage in similar mining operations. Overall, the selection of Plant 2 and Plant 3 ensured a comprehensive and practical understanding of energy consumption patterns, aiding in the development of strategies for reducing energy costs and improving efficiency.

### Carbon pricing scenario analysis

To clarify the financial implications of ASM-related CO_2_ emissions, this study applies a scenario-based carbon pricing approach, using an illustrative value of $24 per ton of CO_2_ from international reports (WorldBank, 2024). Rather than indicating guaranteed revenue, this framework highlights a potential cost or, under specific offset or trading schemes, a cost reduction or limited revenue opportunity if emissions fall below a regulated baseline. In practice, ASM operators would more likely pay for emissions under a carbon tax or cap-and-trade system, rather than earn direct revenue.

$$Revenue \left(USD\right)=C{O}_{2 }Emissions\left(tons\right) x Carbon Price (USD/tons)$$

Annual CO_2_ emissions for each plant and mine were derived using energy consumption data and emission factors aligned with IPCC (2006) guidelines. The primary contributor, electricity, was responsible for over 99% of the total energy consumed, with an emission factor of 25.55 kg CO_2_/GJ. The minimal contributions of diesel and LPG were included to ensure a comprehensive emission profile. Diesel and LPG, which accounted for minimal energy use, were assigned factors of 74.1 kg CO_2_/GJ and 63.1 kg CO_2_/GJ, respectively. The total emissions were calculated as follows:

$$CO_{2}~ Emissions~ \left( {tons} \right) = \mathop \sum \limits_{i = 1}^{n} \left( {E_{i} \times EF_{i} } \right)$$

where

- E_i_ = Energy consumed for each source (electricity, diesel, LPG) in GJ.
- EF_i_ = Emission factor for each energy source (kg CO₂/GJ).

Multiplying total emissions by $24 per ton of CO_2_ yields an approximate carbon cost that ASM operators might incur in a policy environment imposing a price on CO_2_ emissions. While higher carbon prices can motivate cleaner technologies and energy efficiency, the transition can impose financial burdens on ASM operations with limited capital. However, if such systems offer offset credits for verified emission reductions, a portion of this notional cost could transform into limited revenue. Ultimately, this scenario underscores how carbon pricing, whether manifesting as a tax, a trading mechanism, or an offset market can shape ASM decision-making by revealing the economic ramifications of energy use and guiding strategic investments in low-carbon processes.

## Results

Considering the methodology described together with the information obtained, the following main indicators have been calculated (Table 2): Gross Energy Requirement (GER) quantifies the total energy demand in a process or activity, encompassing electricity, fossil fuels, and other sources, providing insights into energy efficiency, environmental impact, and cost-effectiveness (Islam et al., 2020). Treated tonnage gold measures the volume of gold processed to extract gold, reflecting production volume and efficiency, crucial for operational assessment and decision-making in the gold mining industry. Energy Intensity Index (EII) evaluates energy efficiency by comparing energy consumption to output, aiding in identifying optimization opportunities and enhancing operational efficiency across industries (Zhang et al., 2020). GER, treated tonnage gold, and EII collectively offer comprehensive insights into energy dynamics, production efficiency, and environmental impact within mining operations, guiding sustainable resource management and energy efficiency strategies.

**Table 2:** Energy intensity indicators

*Energy intensity indicators*											
Process	Year	GER gross energy requirement GJ	Treated tonnage Au Tm	Treated tonnage per month Tm	Treated tonnage Cu Tm	Treated tonnage per month	Energy Intensity Index per year Au (GJ/Tm)	Energy Intensity Index per year Cu (GJ/Tm)	Energy Intensity per Process per month (GJ/Tm)	Total, CO_2_ Emissions (ton)	Total, CO_2_ Revenue (USD)
Plant 1	2019	9,239,821.67	34,214.48	2,851.21	–	–	6,380.63	–	3,240.67	236,229.47	5,669,507.31
	2020	9,239,821.67	34,214.48	2,851.21	–	–	6,380.63	–	3,240.67	236,269.31	5,670,463.42
	2021	6,080,283.98	45,323.92	3,776.99	–	–	1,367.20	–	1,609.82	155,500.72	3,732,017.23
	2022	9,701,761.74	68,263.99	5,688.67	–	–	2,337.86	–	1,705.45	248,078.34	5,953,880.09
	2023	6,080,283.98	45,323.92	3,776.99	–	–	1,367.20	–	1,609.82	155,525.26	3,732,606.21
Plant 2	2019	15,849,718.38	25,325.00	2,110.42	–	–	12,938.86	–	7,510.23	405,221.09	9,725,306.11
	2020	15,849,718.38	25,325.00	2,110.42	–	–	12,938.86	–	7,510.23	405,289.43	9,726,946.20
	2021	30,813,536.66	–	–	35,660.00	2,971.67	–	29,953.68	–	788,043.30	18,913,039.28
	2022	30,699,513.91	–	–	37,237.00	3,103.08	–	8,033.97	–	785,000.14	18,840,003.43
	2023	15,416,941.46	–	–	35,660.00	2,971.67	–	1,870.63	–	394,344.05	9,464,257.20
Plant 3	2019	1,097,964.00	7,800.00	650.00	–	–	2,369.31	–	1,689.18	28,071.05	673,705.09
	2020	1,097,964.00	7,800.00	650.00	–	–	2,369.31	–	1,689.18	28,075.78	673,818.71
	2021	2,323,785.60	7,984.00	665.33	–	–	5,690.50	–	3,492.66	59,429.84	1,426,316.26
	2022	1,137,279.60	11,000.00	916.67	–	–	1,474.58	–	1,240.67	29,080.74	697,937.81
	2023	2,323,785.60	7,984.00	665.33	–	–	5,690.50	–	3,492.66	59,439.22	1,426,541.36
Plant 4	2019	298,490.40	3,193.00	266.08	–	–	132.99	–	1,121.79	7,631.34	183,152.18
	2020	298,490.40	3,193.00	266.08	–	–	132.99	–	1,121.79	7,632.63	183,183.07
	2021	1,622,007.06	6,140.00	511.67	–	–	14,954.47	–	3,170.05	41,482.15	995,571.64
	2022	3,008,535.80	9,704.00	808.67	–	–	3,763.00	–	3,720.37	76,929.59	1,846,310.17
	2023	1,622,007.06	6,140.00	511.67	–	–	14,954.47	–	3,170.05	41,488.70	995,728.76
Plant 5	2019	8,314,582.13	21,809.53	1,817.46	22,719.76	1,893.31	3,086.89	323.21	4,574.83	212,574.38	5,101,785.06
	2020	8,314,582.13	21,809.53	1,817.46	22,719.76	1,893.31	3,086.89	323.21	4,574.83	212,610.23	5,102,645.43
	2021	8,314,598.45	25,642.12	2,136.84	27,767.92	2,313.99	3,189.54	2,764.31	3,891.07	212,642.38	5,103,417.01
	2022	7,745,666.52	26,900.04	2,241.67	12,034.86	1,002.91	3,315.90	711.99	3,455.31	198,060.12	4,753,442.81
	2023	3,326,542.30	3,070.07	255.84	27,767.92	2,313.99	732.26	1,900.34	13,002.47	85,088.35	2,042,120.48
Mine 1	2019	5,318,751.60	18,631.10	1,552.59	22,719.76	1,893.31	2,095.43	137.93	3,425.72	135,981.62	3,263,558.77
	2020	5,319,921.85	18,631.10	1,552.59	22,719.76	1,893.31	2,095.90	137.97	3,426.48	136,034.47	3,264,827.32
	2021	5,871,015.06	16,787.55	1,398.96	20,912.23	1,742.69	3,848.50	220.58	4,196.69	150,148.75	3,603,570.07
	2022	4,781,458.48	9,304.95	775.41	35,446.92	2,953.91	4,289.25	3,651.51	6,166.34	122,264.01	2,934,336.17
	2023	5,871,015.06	16,787.55	1,398.96	20,912.23	1,742.69	3,848.50	220.58	4,196.69	150,172.45	3,604,138.78
Mine 2	2019	15,845,905.62	18,845.00	1,570.42	–	–	5,860.99	–	10,090.26	405,123.61	9,722,966.62
	2020	15,849,707.59	18,845.00	1,570.42	–	–	5,862.32	–	10,092.68	405,289.15	9,726,939.58
	2021	30,802,369.46	36,710.00	3,059.17	–	–	15,896.17	–	10,068.88	787,757.71	18,906,184.97
	2022	30,795,533.73	5,340.00	445.00	–	–	40,633.51	–	69,203.45	787,455.41	18,898,929.90
	2023	30,802,369.46	36,710.00	3,059.17	–	–	15,896.17	–	10,068.88	787,882.03	18,909,168.70

### Gross energy requirement—GER

From 2019 to 2023, five plants and two mines experienced a 26% increase in their total Gross Energy Requirement (GER), predominantly driven by 23% higher gold production and a 4% rise in copper processing. This jump corresponds to a strong post-COVID production rebound, specifically:

- Mine 2 posted the highest GER, peaking at roughly 30.8 million GJ in 2021 underscoring the intensive energy demands tied to increased output.
- Plant 2 closely followed, more than doubling its GER from about 15.8 million GJ (2019–2020) to 30.7 million GJ (2021–2022), then reverting to 15.4 million GJ by 2023 indicating operational or market adjustments post-peak.
- Mine 2 also mirrored Plant 2’s trajectory running at around 50% capacity during 2019–2020 before consistently operating at full capacity from 2021 onward.

The observed increase in Gross Energy Requirement (GER) across the studied plants and mines from 2019 to 2023 may signify a post-COVID economic recovery trend within the mining sector (Nekhili et al., 2021). As economies rebounded from the pandemic-induced slowdown, demand for commodities like gold and copper likely surged, driving up production volumes and consequently energy consumption. The higher GER reflects heightened industrial activity aimed at meeting the rising demand for these essential metals, which are integral to various sectors including construction, electronics, and renewable energy technologies. The notable increase in gold processing, with an average rise of 23%, could be indicative of renewed investor interest in safe-haven assets amidst economic uncertainties post-pandemic (Gautam et al., 2022). Similarly, the 4% increase in copper processing aligns with the global push for infrastructure development and the transition to cleaner energy sources, driving up demand for copper wiring and components (Roa, 2020) (IRENA, 2023). The energy-intensive nature of mining operations underscores the role of energy efficiency measures and renewable energy integration in ensuring sustainable economic recovery and resilience against future disruptions (Igogo et al., 2021). Therefore, while the GER increase signifies economic recovery, it also highlights the imperative for adopting sustainable practices to mitigate environmental impacts and secure long-term economic stability (Banco Central del Ecuador, 2023).

### Treated tonnage gold

Between 2019 and 2023, the highest Treated Tonnage Gold was recorded at Plant 1 in 2022 (68,263.99 tons), whereas Plant 5 displayed the lowest output (5,340.00 tons) in the same period. Notably, Plant 2 ceased gold processing altogether in 2021, potentially reflecting operational or market-driven constraints. In contrast, for copper, Plant 2 achieved the highest production level (37,237.00 tons) in 2022, while Plant 5 reported a modest 12,034.86 tons that year but recovered to 27,667.92 tons by 2023. These patterns underscore the mining sector’s resilience in the post-COVID environment: Plant 2 focused predominantly on copper over gold, whereas Plant 5 exhibited an inverse production profile. Overall, the data indicate that expanding industrial activity and infrastructure development are driving up demand for both gold and copper, signaling the sector’s capacity to adapt in response to evolving market conditions following the pandemic-induced downturn.

### Energy intensity index—EII

From 2019 to 2023, the Energy Intensity Index (EII) for gold (EII-Au) and copper (EII-Cu) displayed sharp fluctuations, including a 423% increase for gold and a 79% rise for copper. These variations reflect pandemic-related operational shifts and subsequent production increases in plants and mines. For example, Plant 4’s EII-Au soared by 2842%, highlighting major inefficiencies, while Plant 5 balanced reduced GER with a pivot toward copper, suggesting strategic adjustments in response to changing market demands. The relationship between high EII values and elevated CO_2_ emissions underscores the importance of operational optimization.

Energy Intensity per Process per Month (GJ/Tm) further illustrates how resource utilization aligns with greenhouse gas outputs. Mine 2, for instance, recorded extremely high energy intensity and CO_2_ emissions, whereas well-managed plants like Plant 1 maintained more moderate metrics. Such discrepancies highlight the need for advanced technologies, better workflow management, and regulatory frameworks to limit unnecessary energy consumption and reduce emissions.

Though some studies have framed the monetization of CO_2_ reductions as a potential revenue source, many ASM operations remain challenged by capital constraints and lack of formal carbon trading participation. Still, the scenario underscores how adopting cleaner, more efficient processes particularly in grinding can mitigate both energy costs and carbon footprints, aligning economic resilience with sustainability goals.

**Fig. 2:**
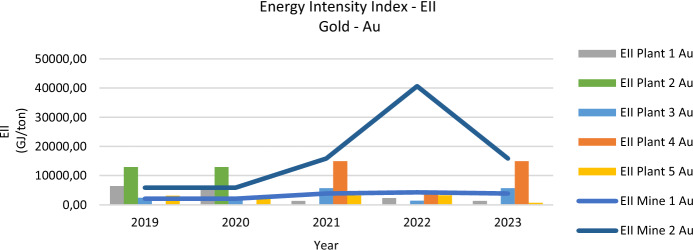
Energy intensity index—gold

Figure 2 illustrates the EII-Au trends, showing an exponential increase for Mine 2 from 2020 onwards, which processed material from various locations due to pandemic-related restrictions. This spike, peaking at 40,633.51 GJ/ton in 2022, underscores the high energy intensity associated with increased gold production during this period. Mine 2’s operational shifts, including owning its benefit plant, contributed to these trends. By 2022, processing shifted back to other plants, normalizing consumption and production patterns.

**Fig. 3:**
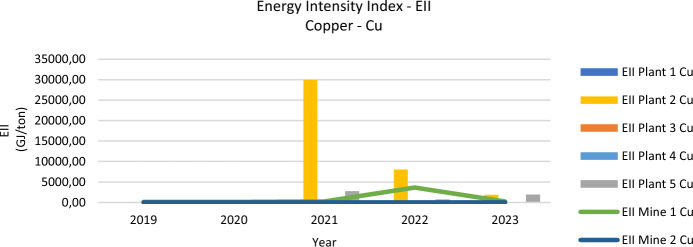
Energy intensity index—copper

Figure 3 depicts the EII-Cu trends, with Plant 2 showing an abnormal peak of 29,953.68 GJ/ton in 2021, aligning with its focus on copper processing. Mine 1’s peak of 3,651.51 GJ/ton in 2022 aligns with gold processing trends, indicating interconnected energy intensity metrics and production strategies.

The analysis of the period 2019–2023 in the Zaruma-Portovelo mining district reveals substantial changes in the Energy Intensity Index (EII) for both gold (EII-Au) and copper (EII-Cu). The EII-Au increased by 423%, while the EII-Cu grew by 79%. These trends reflect the broader economic recovery post-COVID-19 pandemic, where mining operations significantly ramped up production to meet rising global demand.

Data from 2019 to 2023 reveal noteworthy shifts in energy performance across multiple plants and mines, with sharp increases in both the Energy Intensity Index (EII) for gold and copper and higher Gross Energy Requirements (GER). For example, Plant 1 enhanced gold processing efficiency, reporting a 12% decline in EII-Au alongside a 3% drop in GER, whereas Plant 4 experienced severe inefficiency with an EII-Au surge of 2842% and a 121% jump in GER. Such divergences underscore how targeted energy management can improve outcomes, while poor practices exacerbate consumption.

The post-COVID period also saw drastic variations in energy use. Plants like Plant 2 achieved reductions in EII-Au (25%) and EII-Cu (37%) but faced an 11% GER increase, implying scale-up or operational inefficiencies. Meanwhile, Plant 5 reduced its energy intensity for gold by 18% but posted a 212% rise for copper, reflecting a strategic focus on copper that heightened resource demands in that segment. Mines followed similar patterns: Mine 2 noted a 66% increase in EII-Au and 24% in GER, mainly driven by expanded production capacity and interregional ore processing.

Comparisons of EII, CO₂ emissions, and monthly process-level energy intensities provide further insight: Mine 2 exhibited exceptionally high values in 2022 (EII-Au of 40,633.51 GJ/ton), correlating with elevated CO_2_ outputs, whereas better-managed facilities like Plant 1 maintained more moderate intensities and emissions. The data thus highlights the critical role of technological upgrades, optimized workflows, and robust oversight in curbing excessive energy use and associated environmental impacts.

Table 3, CO_2_ Emission per Year presents the annual contribution of different energy sources GLP (Liquefied Petroleum Gas), Electricity, and Diesel Oil to CO_2_ emissions per gigajoule (kg CO_2_/GJ) for the period 2019 to 2023 based on IPCC methodology (IPCC, 2006). Electricity emerges as the dominant contributor to CO_2_ emissions, maintaining a consistently high value around 25.55 kg CO_2_/GJ, reflecting the energy matrix's heavy reliance on electricity as the primary energy source. Diesel Oil, while contributing significantly less, shows slight fluctuations, with values ranging from 0.0086 to 0.0283 kg CO_2_/GJ, indicating operational variations in diesel consumption. GLP contributes minimally to CO_2_ emissions, with its values decreasing from 0.00139 kg CO_2_/GJ in 2019 to 0.00013 kg CO_2_/GJ in 2023, signifying a reduced dependence on GLP over the years. The Total CO_2_ Emission per GJ remains remarkably stable across the years, averaging 0.02557 tons CO_2_/GJ, showing consistency in the district's overall CO_2_ emission profile despite operational and energy source variations.

**Table 3:** CO_2_ emission per year

*CO*_*2*_* emission per year*				
Year	GLP CO_2_ (kg/GJ)	Electricity CO_2_ (kg/GJ)	Diesel oil CO_2_ (kg/GJ)	Total CO_2_ (ton/GJ)
2019	0.00139	25.5565	0.0086	0.0255665
2020	0.00139	25.5542	0.0152	0.0255708
2021	0.00067	25.5523	0.0217	0.0255746
2022	0.00013	25.5545	0.0158	0.0255704
2023	0.00013	25.5502	0.0283	0.0255786
Average 2019–2023	0.00074	25.55352	0.01791	0.02557

It is important to note that the largest source of energy for the processing of minerals comes from electricity as evidenced in Table 4, the energy consumed by the plants and mines studied is 99.9% electricity, however, there is a minimum consumption of Diesel and LPG that are used for auxiliary services.

**Table 4:** Energy mix

Year	LPG %	Electricity %	Diesel oil %	Total
2019	0,0022	99,9862	0,012	100
2020	0,0022	99,9773	0,020	100
2021	0,0011	99,9697	0,029	100
2022	0,0002	99,9784	0,021	100
2023	0,0002	99,9616	0,038	100
Average 2019–2023	0,0012	99,9747	0,024	100

### Energy consumption in intensive processes—ECMP

Table 5 presents a detailed analysis of energy consumption by process at points of highest consumption in two mining plants, Plant 2 and Plant 3, measured in Giga–Joules (GJ) over 20 working days. The table disaggregates the data into four primary processes: crushing, grinding, flotation, and other activities, highlighting daily variations in energy usage. Grinding consistently stands out as the most energy-intensive process, accounting for the largest share of consumption across both plants, which aligns with the high energy demands typically associated with gold reduction in mining operations.

**Table 5:** Energy consumption by mining process (ECMP)

Day	Plant 2 (GJ)				Plant 3 (GJ)			
	Crushing	Grinding	Flotation	Other	Crushing	Grinding	Flotation	Other
1	0.38	12.06	2.25	3.91	0.74	2.66	0	0.92
2	0.59	21.61	1.45	6.29	1.53	5.50	0	1.89
3	0.54	19.45	3.23	6.17	0.97	3.47	0	1.20
4	0.78	22.06	0.97	6.33	0.96	3.44	0	1.18
5	0.76	22.24	5.01	7.45	1.42	5.10	0	1.76
6	0.75	22.71	5.08	7.59	1.78	6.40	0	2.20
7	0.71	22.60	5.05	7.54	1.43	5.12	0	1.76
8	0.66	22.53	5.05	7.51	1.94	6.94	0	2.39
9	0.77	22.40	4.99	7.48	1.63	5.84	0	2.01
10	0.64	22.66	5.01	7.52	1.49	5.36	0	1.85
11	0.64	22.47	5.05	7.49	1.97	7.07	0	2.43
12	0.80	22.34	5.10	7.51	1.69	6.06	0	2.08
13	0.77	22.21	5.12	7.47	1.69	6.06	0	2.09
14	0.69	21.87	5.18	7.38	1.73	6.21	0	2.14
15	0.57	16.03	5.12	5.77	1.23	4.40	0	1.52
16	0.60	12.43	5.19	4.84	1.51	5.42	0	1.87
17	0.53	10.39	5.08	4.25	1.70	6.08	0	2.09
18	0.50	12.57	5.13	4.84	1.79	6.42	0	2.21
19	0.63	12.54	5.14	4.87	1.90	6.81	0	2.34
20	0.67	12.41	5.11	4.83	0.51	1.83	0	0.63

In Plant 2, energy consumption for grinding often exceeds 21.6 GJ per day, while other processes, such as crushing and flotation, consume considerably less, indicating that grinding is the primary driver of energy costs. Similarly, in Plant 3, grinding remains the dominant process, albeit with slightly lower values than in Plant 2. Interestingly, the others category shows significant energy usage in both plants, suggesting that auxiliary activities and equipment, which may include pumping, ventilation, or material handling, also contribute substantially to overall consumption. This emphasizes the need to focus on both core and supporting processes when seeking energy efficiency improvements.

The data from Table 5 reveals clear patterns that can guide energy efficiency strategies. Given the high energy demand of grinding, efforts to optimize this process, such as implementing more efficient grinding technologies or adjusting operational parameters, could yield significant energy savings. Additionally, the notable energy use in the others category suggests opportunities to improve auxiliary systems' efficiency. Overall, Table 5 serves as a reference for identifying energy-intensive areas within the mining plants, providing valuable insights for developing targeted strategies to reduce energy consumption and enhance sustainability in mining operations.

## Discussion

The period from 2019 to 2023 in the Zaruma Portovelo mining district has provided substantial insights into the dynamics of energy consumption and efficiency in gold and copper mining operations, particularly in the context of the post-COVID-19 economic recovery. The Gross Energy Requirement (GER) saw a significant 26% increase, highlighting heightened energy demands in line with increased production activities. Gold processing experienced an average increase of 23%, while copper processing grew by 4%, reflecting a robust recovery in mining activities as global demand for these commodities surged post-pandemic.

Overall, the GER, EII-Au, EII-Cu, and treated tonnage metrics collectively reveal the post-pandemic recovery trends in the Zaruma Portovelo mining district. The substantial increases in energy intensity and gross energy requirements align with heightened production efforts to meet rising demand for gold and copper. The data underscores the critical need for improved energy efficiency and sustainable practices to mitigate environmental impacts and ensure long-term economic stability. The mining sector's ability to adapt and recover post-pandemic highlights its pivotal role in the broader economic recovery and the importance of strategic energy management in achieving sustainable growth.

Comparisons between our findings and prior studies underscore both the consistency of gold production and the emerging role of copper in Portovelo-Zaruma’s artisanal and small-scale mining (ASM). Although Gonçalves et al. (2017) documented approximately 1.7–1.9 million tons of ore processed annually (yielding an estimated 7–8 tons of gold in 2015) across 87 units from 2013 to 2015, our narrower sample from five processing plants and two mines between 2019 and 2023 captured a total of 3.08 tons of gold and 122,503.22 tons of copper, with mean annual outputs of 0.62 tons and 24,500.64 tons, respectively. Differences in timeframes and sample sizes notwithstanding, the data reveal sustained interest in gold extraction and a growing emphasis on copper, which demands targeted measures ranging from optimized processing and technological upgrades to expanded training to bolster productivity while limiting environmental footprints. Achieving such improvements requires policy incentives, capital investment, and cross-sector collaboration, particularly as rising copper production alters infrastructure needs and may intensify energy consumption. Ensuring transparent reporting and robust data collection can further support evidence-based strategies, fostering broader sustainability outcomes and reinforcing the industry’s economic contributions in post-pandemic contexts.

Observed discrepancies in energy intensity across different ASM operations or countries can be attributed to multiple interconnected factors, including variations in processing technologies, regulatory frameworks, mining techniques, and the availability and type of energy sources (Famiyeh et al., 2021). Advanced processing technologies generally result in reduced energy consumption per unit of material processed, whereas less efficient practices or outdated equipment can significantly elevate energy intensity (Tomassi, 2024). Institutional pressures, as outlined by coercive (regulatory), mimetic (competitive imitation), and normative (industry standards and social expectations) factors, substantially influence the adoption of sustainability practices across economic, environmental, and social dimensions (Li et al., 2023). Specifically, strong regulatory frameworks drive operational efficiencies by enforcing compliance with stringent environmental and energy-use standards. Additionally, variations in mining techniques, such as differences in gold extraction and benefit processes, markedly affect energy requirements. Finally, the availability and choice of energy sources, whether renewable or fossil-based, directly impact overall energy efficiency and sustainability outcomes (Abbaspour & Abbaspour, 2022). Understanding these diverse institutional and technical factors is essential for policymakers and industry leaders to develop targeted strategies aimed at reducing energy intensity and advancing comprehensive sustainability practices within the ASM sector (Finn et al., 2024).

Each plant, operating as a private entity, submits data regarding the quantity of gold processed to the Mining Regulation and Control Agency (ARCOM). ARCOM then compiles this data without independent verification, relying solely on plant owner submissions, which can result in discrepancies. This reporting process is closely associated with electricity consumption billing, recorded by the local electricity provider, CNEL. Consequently, monitoring energy usage primarily depends on electricity bills and tariffs. However, gold processing records may contain inaccuracies or deliberate omissions, potentially influenced by the regulatory framework governing these operations. The study acknowledges the inherent risks associated with self-reported data but does not clearly propose solutions. Therefore, implementing explicit recommendations for independent verification or enhanced regulatory oversight is necessary to ensure accurate, transparent reporting of energy consumption in the ASM sector. Recommended approaches for verification include regular audits by independent third parties, cross-referencing gold processing data with satellite imagery or drone surveillance, and incorporating real-time monitoring technologies to track energy consumption and operational efficiency more accurately (McKay, 2025). Recognizing and addressing these institutional and technical factors is crucial for policymakers and industry stakeholders aiming to formulate effective strategies that lower energy intensity and foster comprehensive sustainability within the ASM sector.

According to Marshall et al. (2020) the Puyango-Tumbes River region in Ecuador hosts 87 gold processing centers, most of which operate in Portovelo—Zaruma. These facilities handle gold from various mines and artisanal gold miners, with over 70% of the local workforce engaged in artisanal mining producing an estimated 40–80 tons of gold every 14–21 days. Typically, miners rely on the centers for grinding and amalgamation services, while cyanidation circuits are used by processing center owners to recover additional gold from tailings. In our analysis (2019–2023), the five plants and two mines studied each processed, on average, 1,535.00 tons of gold and 820 tons of copper per month, amounting to 219.3 tons per plant or mine monthly. Such high throughput underscores both the centrality of the state’s role in guiding extractive activities and the importance of aligning energy consumption strategies with fiscal objectives. By leveraging the economic value generated by these processing operations, public policies can bolster state revenue while advocating more efficient, less environmentally harmful practices in gold-processing facilities.

The analysis by Jones et al. (2021) projects a notable surge in global copper demand by 2030, largely driven by the post-COVID-19 rebound in economic activities, expanding electric mobility initiatives, the energy transition, and the growing need to increase renewable energy capacity. In line with this trend, Roa (2020) reports that the average price of copper rose to US$4.60 per pound in 2021 and US$4.87 per pound in 2022, mirroring the amplified production and energy usage observed at Mine 1 (35,447 tons treated) and Plant 2 (37,237 tons treated) in 2022. This correlation underscores how supply–demand dynamics enable industries to adjust their output for optimal profitability, highlighting the interplay between market forces, industrial operations, and associated environmental impacts in artisanal and small-scale mining contexts.

The methods proposed for evaluating energy consumption and energy intensity index (EII) in relation to the quantity of treated tonnage of gold processed in Zaruma have demonstrated their efficacy as a direct approach for determining the extent of gold processing and its associated energy consumption. In this sense, our work applies the methodology proposed by Aramendia et al. (2023) that was developed to carry out energy consumption scenarios in the mining industry over time., this methodology takes into account the energy requirements of mining activities, both direct and indirect, which are quantified using Life Cycle Analysis methods. Direct energy requirements refer to the energy used in situ to operate the mine, while indirect energy requirements are used ex-situ in the mine supply chain to provide inputs like chemicals and machinery. The Gross Energy Requirement (GER) is obtained by adding both direct and indirect energy requirements. As both direct and indirect energy requirements are likely to increase due to mineral depletion, the GER is used to measure the primary energy intensity of each mineral. By employing this methodology, we were able to assess the correlation between the rise in mineral demand and the drop in energy intensities, resulting in an anticipated increase in the industry's overall energy consumption.

Katta et al. (2020) provide a useful comparative benchmark by reporting energy intensities of 0.7, 149.8 × 103, and 1.8 GJ/t for iron, gold, and potash mining in Canada, respectively, accompanied by corresponding greenhouse gas (GHG) emissions of 33, 4922 × 103, and 158 kg CO_2_ eq./Mg. Their findings emphasize the disproportionately high energy requirement for gold extraction (149,800 GJ/t), mirroring the pronounced Energy Intensity Index (EII) of 5,970.90 GJ/t observed in Zaruma—Portovelo gold mining operations. Such convergence indicates that gold mining presents a significant challenge in both Canadian and Ecuadorian contexts, where elevated energy consumption drives considerable GHG emissions. Consequently, this underscores the urgency of implementing energy-efficient technologies ranging from optimized grinding processes to advanced tailings management to curtail the environmental footprint of gold mining. By prioritizing technological improvements and robust policy measures, stakeholders can reduce energy intensity while mitigating adverse ecological impacts.

The data presented in Energy consumption by mining process (ECMP) highlights that grinding is consistently the most energy-intensive process in both Plant 2 and Plant 3, accounting for the largest share of energy consumption. This aligns with findings in the literature, which identify grinding as a major contributor to energy use in mining operations (Jeswiet & Szekeres, 2016). The significant energy demand for grinding in these plants suggests that this stage offers the greatest potential for energy efficiency improvements. Implementing advanced grinding technologies or optimizing operational parameters could reduce energy consumption, leading to cost savings and a smaller environmental footprint.

Additionally, the "Other" category of energy consumption indicates substantial energy use in auxiliary processes such as pumping, ventilation, or material handling. This suggests that beyond the core mining processes, there is room for improving energy efficiency in supporting systems. Addressing inefficiencies in these auxiliary activities can provide further opportunities for reducing overall energy consumption, thus enhancing the sustainability of mining operations. Therefore, a comprehensive energy management approach that considers both primary and auxiliary processes is essential for achieving significant improvements in energy efficiency in these ASM processing plants.

The data in Energy Consumption by Mining Process ECMP, reveals grinding as the most energy-intensive process, aligns closely with the calculated GER and EII values over the period from 2019 to 2023. For instance, Plant 2 exhibited the highest GER among the studied plants, with values reaching up to 30,699,513.91 GJ in 2022. This high GER correlates with the consistently elevated energy consumption for grinding in ECMP, often exceeding 6 MWh per day. This supports the idea that grinding is a dominant driver of energy consumption in small-scale mining operations, as it is responsible for the reduction of gold into finer particles, which inherently requires substantial energy input. Therefore, any attempts to improve energy efficiency should prioritize optimizing the grinding process, as it offers the greatest potential for reducing overall energy usage.

The relationship between Energy Intensity per Process per Month (GJ/Tm) and Total CO_2_ Emissions highlights how inefficiencies in energy distribution directly amplify environmental impacts. In Plant 4 (2021), an Energy Intensity per Process of 3,170.05 GJ/Tm correlated with an EII-Au of 14,954.47 GJ/Tm and Total CO_2_ Emissions of 41,482 tons, demonstrating a clear link between increased energy usage per process unit and higher emissions. Similarly, Plant 2 (2021), with an Energy Intensity per Process per Month of 7,510.23 GJ/Tm, reported Total CO_2_ Emissions of 788,043 tons, reinforcing the environmental costs of elevated energy demands.

Interestingly, facilities that maintained moderate Energy Intensity per Process metrics, such as Plant 3 (2022) with 1,240.67 GJ/Tm, managed to stabilize their GER and limit emissions growth (29,080 tons CO_2_). This highlights the potential for energy efficiency measures, such as equipment optimization and better scheduling, to minimize energy requirements without compromising production volumes. High Energy Intensity per Process per Month (GJ/Tm) often signals systemic inefficiencies, operational mismanagement, or aging infrastructure. Conversely, lower energy intensity values are typically associated with optimized production systems, better energy distribution, and reduced environmental impacts.

The treated tonnage and the Energy Intensity Index (EII) relationship is also crucial to understanding the broader implications of the data in Table 4. For example, the EII for gold (EII-Au) and copper (EII-Cu) reached averages of 5970.90 GJ/ton and 1435.71 GJ/ton, respectively, over the study period. These data highlight the substantial energy required to process each ton of mineral, emphasizing the energy-intensive nature of ASM operations. The significant energy usage captured in ECMP, especially for grinding, contributes directly to these high EII values. By focusing on energy efficiency measures such as more efficient grinding technologies, reducing idle times, or optimizing operational settings, there is a clear opportunity to decrease both the EII and overall GER. Ultimately, this can lead to more sustainable mining practices and lower operational costs, which are crucial for the long-term viability of ASM in the region.

Moreover, the auxiliary energy consumption categorized under "Other" processes in ECMP points to additional inefficiencies in energy management within these operations. Although not as energy intensive as grinding, these auxiliary processes significantly contribute to the total energy consumption profile. Their impact on the GER and EII suggest that improving energy efficiency must go beyond the primary processes and address the entire energy usage spectrum within ASM plants. This holistic approach to energy management would not only reduce the overall GER and EII but also enhance the sustainability and economic competitiveness of small-scale mining operations in Ecuador. Therefore, the integration of energy-efficient practices, technologies, and strategic interventions in both primary and auxiliary processes can play a critical role in achieving energy sustainability in the ASM sector.

The artisanal and small-scale mining (ASM) sector in Ecuador generates significant revenue from CO_2_ emissions trading, highlighting a potential financial mechanism to drive investments in cleaner technologies. Between 2019 and 2023, CO_2_ revenues across multiple ASM processing plants reflected substantial monetary returns linked to carbon credits and emission offsets (Missbach et al., 2024; Saka et al., 2024; Wang et al., 2023; Zhang et al., 2022). However, despite these financial gains, a limited portion of this revenue has been reinvested into energy-efficient technologies or sustainable practices. This disconnect raises critical concerns about the long-term sustainability and environmental responsibility of ASM operations.

Investing in clean technologies such as advanced grinding systems, hybrid electric machinery, and renewable energy sources (e.g., solar or hydroelectric solutions) presents a viable pathway to reduce both carbon emissions and energy intensity (Abbaspour & Abbaspour, 2022; Jacob & Müller, 2022; Østergaard et al., 2020). For instance, grinding and comminution stages account for up to 70% of total energy consumption, representing a clear opportunity for technological optimization. The integration of variable frequency drives (VFDs), energy management systems, and automated process controls could drastically reduce energy waste while enhancing operational efficiency.

Moreover, redirecting CO_2_ revenues towards research and development (R&D) in sustainable mining technologies could create a positive feedback loop. With financial support from carbon credit revenues, ASM facilities can pilot new technologies, train operators, and gradually phase out inefficient, high-emission machinery. This strategy aligns with global sustainability goals and offers a dual benefit: reducing environmental impact and improving profitability through long-term operational savings.

However, barriers such as regulatory challenges, lack of technical expertise, and initial capital investment requirements must be addressed to enable this transition. Collaborative efforts involving government incentives, international funding mechanisms, and public–private partnerships are essential to create an enabling environment for technology adoption (Keane et al., 2023; Zharan & Bongaerts, 2017). Furthermore, implementing transparent tracking systems for CO₂ revenue allocation can ensure accountability and maximize the sector's contribution to Ecuador's sustainable development agenda.

## Conclusions

The period from 2019 to 2023 in the Zaruma Portovelo mining district revealed significant insights into the energy consumption and efficiency dynamics of gold and copper mining operations, especially in the post-COVID-19 economic recovery context. Gold processing grew by an average of 23%, and copper processing by 4%, reflecting a strong recovery as global demand for these commodities surged. These metrics underscore the critical need for improved energy efficiency and sustainable practices to mitigate environmental impacts and ensure long-term economic stability. Despite the sector's robust post-pandemic recovery, highlighted by increased GER and EII metrics, inefficiencies and outdated technologies remain challenges, particularly in gold processing.

The treated tonnage of gold and copper, along with the Energy Intensity Index (EII) for both metals, provides a comprehensive picture of the efficiency and intensity of mining operations. Plant 1 demonstrated improved energy efficiency with a 12% decrease in EII-Au and a 3% decrease in GER, indicating more efficient gold processing without copper involvement. Plant 2 exhibited inefficiencies, with EII-Au and EII-Cu decreasing by 25% and 37%, respectively, but GER increasing by 11%. This suggests that despite lower energy intensity, the overall energy consumption rose due to increased production volumes or operational inefficiencies. Plant 3 performance showed an 88% increase in EII-Au and a 41% rise in GER, indicating higher energy consumption per unit of gold produced, pointing to decreased operational efficiency. Plant 4 presented extreme inefficiency, with a 2842% increase in EII-Au and a 121% rise in GER, possibly due to outdated technology or poor management practices. Plant 5 shifted its focus towards copper processing, as evidenced by an 18% decrease in EII-Au and a 212% increase in EII-Cu, coupled with a 16% reduction in GER. This indicates a strategic realignment towards copper, managing to reduce overall energy consumption while focusing on copper production. Mine 1 showed significant increases in both EII-Cu (380%) and EII-Au (21%), with a 4% rise in GER, reflecting intensified production efforts and increased energy consumption for copper processing. Mine 2 66% rise in EII-Au and 24% increase in GER highlights its role in processing materials from various regions, leading to higher energy consumption and operational expansion post-pandemic.

This trend is further underscored by the exponential increase in EII-Au for Mine 2 from 2020 onwards, peaking at 40,633.51 GJ/ton in 2022. Such spikes illustrate the high energy intensity linked to the increased gold production during the recovery period. Plant 1 maximum treated tonnage of gold in 2022 and Plant 2's highest copper processing values in the same year reflect a significant rebound in production activities. Plant 2 cessation of gold processing in 2021 and subsequent recovery in copper processing highlight the operational adjustments in response to market demands and pandemic challenges. The recovery in Plant 5 copper processing from 2022 to 2023 further signifies the sector's capacity to navigate economic uncertainties and drive post-pandemic recovery efforts.

The study found significant variability in energy efficiency across different plants, with some showing improved energy efficiency and others displaying substantial increases in energy intensity. These findings underscore the necessity of strategic energy management and the implementation of energy-efficient technologies to promote sustainable growth. The sector's adaptability and resilience were evident, but the high energy intensity associated with gold mining, both locally and globally, highlights the urgent need for energy-efficient practices to reduce environmental impacts and enhance sustainability in ASM operations.

The analysis of the Zaruma-Portovelo mining district (2019–2023) reveals a direct correlation between CO_2_ emissions and Energy Intensity Indicators (EII), particularly in facilities with high Energy Intensity per Process per Month (GJ/Tm). For example, Plant 4 (2021) and Mine 2 (2022) reported extreme energy intensity values (3,170.05 GJ/Tm and 69,203.45 GJ/Tm) and correspondingly high Total CO_2_ Emissions (41,482 tons and 787,455 tons). Electricity, contributing an average of 25.55 kg CO_2_/GJ, remains the dominant driver of emissions. While Total CO_2_ Emissions per GJ (0.02557 ton CO_2_/GJ) remained stable across the years, localized spikes highlight the inefficiencies tied to energy-intensive processes, especially in copper processing. This underscores the need for targeted energy efficiency improvements and technological upgrades to reduce emissions effectively.

The correlation between CO_2_ emissions, Gross Energy Requirement (GER), and Energy Intensity Index (EII) emphasizes the critical role of energy management strategies. Facilities like Plant 1 achieved controlled Energy Intensity per Process (1,609.82 GJ/Tm) and lower Total CO_2_ Emissions (155,525 tons), showcasing effective energy-saving measures. In contrast, plants with rising GER but stagnant efficiency, such as Plant 2 (2021) and Mine 2 (2022), experienced sharp increases in emissions. Given that electricity accounts for nearly 99.97% of the energy mix, inefficiencies directly amplify CO_2_ emissions.

Grinding is the most energy-intensive process within the ASM plants, significantly contributing to the overall energy consumption reflected in the Gross Energy Requirement (GER) and Energy Intensity Index (EII). Therefore, targeted interventions in grinding processes, such as the adoption of advanced grinding technologies or optimization of operational parameters, can lead to substantial reductions in energy usage. Implementing these improvements could result in significant cost savings and contribute to reducing the environmental impact of ASM operations.

Beyond the core processes, the considerable energy consumption observed in auxiliary activities, categorized as "Other" in ECMP, indicates that energy efficiency efforts should not be limited to primary processes like grinding. A holistic energy management strategy that addresses both core and supporting activities is essential for achieving meaningful improvements in energy efficiency. This approach would help reduce the overall GER and EII, ultimately fostering more sustainable and economically viable ASM operations in the Zaruma-Portovelo mining district and similar contexts.

The data reveals that increases in treated tonnage, particularly in gold and copper production, are directly associated with higher Energy Intensity Index (EII) values, indicating that energy consumption escalates as production scales up. This trend underscores the importance of implementing energy efficiency measures as production expands, ensuring that the benefits of increased output are not offset by disproportionate rises in energy consumption. Addressing energy efficiency proactively as production grows will be crucial for maintaining sustainable and cost-effective operations in ASM plants.

The study's findings indicate that outdated equipment and inefficient processes are significant contributors to high energy consumption in ASM operations. Investing in modern, energy-efficient technologies, particularly for grinding and auxiliary processes, presents an opportunity to substantially lower the Gross Energy Requirement (GER) and EII across plants. By prioritizing technological upgrades, ASM operations can achieve a more sustainable balance between productivity and energy consumption, which is vital for the long-term viability and environmental sustainability of the sector.

Artisanal and small-scale mining (ASM) sector faces significant challenges in energy efficiency and carbon emissions reduction, despite generating substantial revenue from CO₂ emissions trading. However, limited reinvestment of these funds into clean technologies hinders progress. Key opportunities lie in adopting energy-efficient grinding systems, hybrid electric machinery, and renewable energy sources to reduce energy intensity and emissions. Redirecting CO₂ revenues towards technology adoption, workforce training, and research is essential for long-term sustainability. Achieving this transformation requires stronger regulatory frameworks, financial incentives, and collaborative partnerships. By effectively utilizing CO₂ revenue as a driver for clean technology adoption, the ASM sector can align economic growth with environmental responsibility, setting a benchmark for sustainable mining practices.

This study advances existing knowledge on energy efficiency and carbon footprints within artisanal and small-scale mining (ASM) operations in Ecuador, highlighting significant opportunities for policy action. The following three key policy recommendations emerge: Implement robust independent verification systems, including regular third-party audits and remote sensing technologies, to ensure the accuracy and transparency of energy consumption and emissions reporting. Promote the widespread adoption of energy-efficient technologies and optimized energy management practices, particularly in energy-intensive processes like grinding, to substantially reduce energy use and carbon emissions. Enhance regulatory frameworks by providing incentives and targeted support for integrating renewable energy solutions and cleaner technologies, facilitating sustainable growth within the ASM sector while aligning economic and environmental goals.

## Data Availability

No datasets were generated or analysed during the current study.
